# Descriptive epidemiology of prevalence of exercise habits among participants with hypertension: The National Health and Nutrition Survey 2013–2018

**DOI:** 10.1002/jgf2.683

**Published:** 2024-03-01

**Authors:** Noritoshi Fukushima, Shiho Amagasa, Hiroyuki Kikuchi, Susumu S. Sawada, Masaki Machida, Shigeru Inoue

**Affiliations:** ^1^ Department of Preventive Medicine and Public Health Tokyo Medical University Tokyo Japan; ^2^ Graduate School of Public Health Teikyo University Tokyo Japan; ^3^ Faculty of Sport Sciences Waseda University Tokorozawa Japan

**Keywords:** blood pressure, exercise therapy, guidelines adherence, management, physical activity

## Abstract

**Background:**

The current Japanese hypertension management guidelines (2019) recommend regular exercise for all patients with hypertension. However, limited evidence is available regarding the prevalence of exercise habits in these patients. Therefore, we examined the proportion of participants who met the recommendations on exercise in the Japanese hypertension management guidelines (2019) using a nationally representative sample.

**Methods:**

Participants aged ≥20 years from the Japanese National Health and Nutrition Examination Survey conducted from 2013 to 2018 were included. Participants with hypertension were defined as those with blood pressure level ≥140/90 mmHg or those who used antihypertensive drugs. Adherence to the guideline recommendations, stratified by gender, age category, blood pressure level, and medication status, was examined.

**Results:**

This study included 13,414 participants with hypertension (age 68.2 ± 11.7 years, 48.1% men). Among them, 31.8% of participants with hypertension (36.8% of men and 27.3% of women) met the guidelines. Regarding age, the 20–64 years age group had the lowest proportion of patients who met the guidelines (22.4%), followed by those in the 65–74 (37.7%) and ≥75 years age groups (34.5%). Adherence to the guidelines did not significantly differ according to blood pressure levels (<120/<80, 120–129/<80, 130–139/80–89, 140–159/90–99, and 160–179/100–109 mmHg) and presence of antihypertensive medications.

**Conclusion:**

One‐third of participants with hypertension engaged in exercise as recommended by the current hypertension management guidelines. Promotion of exercise therapy and monitoring exercise habits among participants with hypertension is warranted.

## 1 INTRODUCTION

Exercise plays an important role in reducing blood pressure (BP)^1^, ^2^, ^3^ through pathophysiological improvements, such as alteration of vascular structure (remodeling and rarefaction) and attenuated oxidative stress in vascular endothelial function.^4^ Regular exercise has been widely recognized as an effective therapy for reducing the number of antihypertensive medications^2^ and preventing cardiovascular diseases among patients with hypertension (HTN).^4^, ^5^ Therefore, the current Japanese HTN management guidelines (2019) recommend that all patients with HTN, except those with grade III HTN, should exercise for at least 30 min daily or 180 min weekly.^6^ Exercise therapy is currently expected to be implemented for HTN management^6^, ^7^; however, the actual proportion of patients with HTN who engage in regular exercise remains unclear. A high proportion of patients with HTN who regularly exercise suggests that the current clinical guidelines are effectively promoted and that the healthcare system is functioning well.^8^ Conversely, a low proportion may indicate inadequate promotion of guidelines; insufficient content in the guidelines; or potential issues with the HTN healthcare system, including those related to reimbursement. In this case, it may be necessary to invest in new strategies and resources to encourage exercise therapy.^9^ Since HTN is the most prevalent condition among outpatients in Japan,^10^ understanding the prevalence of exercise habits among patients with HTN is essential for improving HTN management and informing future revisions of HTN guidelines.^6^

Several studies have reported the prevalence of regular exercise among patients with HTN^11^, ^12^, ^13^; however, these studies had small sample sizes and were limited to patients in low‐ and middle‐income countries, including Ethiopia, Iran, and China.^11^, ^12^, ^13^ Moreover, medical resources, healthcare systems, and treatment strategies available in low‐ and middle‐income countries differ from those in high‐income countries.^5^, ^9^, ^14^, ^15^, ^16^ Therefore, exercise habits in patients with HTN may differ among low‐, middle‐, and high‐income countries. Hence, it is crucial to assess the proportion of patients engaging in exercise in each country in order to enhance the approach to exercise therapy in the healthcare system.

In Japan, the National Health and Nutrition Survey (NHNS‐J) provides data on exercise habits and the use of antihypertensive medications in a representative sample of the population.^17^ Therefore, this study aimed to examine the proportion of participants who performed exercise as recommended by HTN management guidelines, using a representative sample of Japanese participants stratified according to gender, age group, BP level, and medication status.

## 2 METHODS

### 2.1 The National Health and nutrition survey of Japan (NHNS‐J)

The NHNS‐J, a cross sectional household interview and examination survey conducted each November, has been conducted annually since 1945 by the Ministry of Health, Labor, and Welfare (MHLW) under the Health Promotion Law of Japan. This survey comprised three parts: (1) physical examination, (2) nutritional aspects, and (3) lifestyle. Detailed aspects of the surveys have been described elsewhere.^18^ From 2013 to 2018, NHNS‐J data on BP was obtained using a consistent BP measurement method; moreover, the same questionnaire on exercise habits was administered to participants each year. Therefore, we used raw data from the physical examinations in the NHNS‐J survey from 2013 to 2018 to ensure data consistency.

### 2.2 Participants

In the NHNS‐J survey, individuals aged ≥20 years were invited to participate in the physical examination component of the survey, which comprised BP measurement and medical interviews, including exercise habits, in the presence of healthcare workers. All participants aged ≥20 years who consented to participate in this part of the survey were considered eligible for the present study. Participant sampling designs for the NHNS‐J have been described elsewhere.^18^ Briefly, every year (except for the survey conducted in 2016), the NHNS‐J began with the selection of 300 census units, which were randomly chosen from census enumeration areas previously selected across Japan as part of the Comprehensive Survey of Living Conditions of the People on Health and Welfare. Each census unit included ≈20 households; therefore, 5000–6000 households were sampled annually. Expanded surveys in 2016 were implemented using a stratified single‐stage cluster sample design to compare the NHNS‐J results across 47 prefectures in Japan. For the 2016 survey, 24,187 households from 475 census units were chosen, comprising 10 areas from each prefecture (15 areas in Tokyo). Selected households whose head was not Japanese were excluded from the survey. Moreover, the following participants were excluded from the analyses: those without data on BP, exercise habits, or antihypertensive medication use; pregnant women and nursing mothers; those who were prohibited from exercising by physicians; and participants with degree III HTN (i.e., systolic BP ≥180/diastolic BP ≥110 mmHg).^6^

### 2.3 Data collection

Data regarding BP, weight, height, and exercise habits were obtained from the annual records of physical examinations in the NHNS‐J. Physical examinations, including medical interviews, were performed by physicians or public health nurses at designated community centers.^17^ Antihypertensive drug use was recorded during medical interviews.^17^ Information regarding medication status for dyslipidemia (DLP) and diabetes mellitus (DM) was also obtained.^17^ Body mass index (BMI) was calculated by dividing the weight in kilograms by height in meters squared (kg/m^2^) and categorized as BMI <25 or BMI ≥25.^19^

### 2.4 Blood pressure measurement

BP was measured twice by the medical staff using a Riva–Rocci Mercury Sphygmomanometer after the participants rested for at least 5 min in a sitting posture, in accordance with the instructions of the NHNS‐J.^17^ A pressure cuff was applied to the right arm, and the arm cuff position was maintained at heart level. Medical staff were instructed to deflate the mercury column at 2 mmHg/s and to record the first and last audible Korotkoff sounds as systolic and diastolic BPs, respectively.^17^ The mean of the two BP measurements was used for the analysis. BP values were categorized as <120/80 mmHg, 120–129/80, 130–139/80–89, 140–159/90–99, and 160–179/100–109 mmHg, in accordance with the HTN management guidelines.^6^, ^7^ Participants with HTN were defined as those with a mean systolic BP ≥140 mmHg and/or diastolic BP ≥90 mmHg and/or antihypertensive drug use.^6^, ^7^

### 2.5 Evaluation of exercise habits

Physicians and public health nurses asked the participants whether they were prohibited from exercising by physicians.^17^ Participants who were not prohibited from exercising were subsequently asked about the frequency (days per week) and exercise duration per session. Information on exercise was measured through the following questions of the NHNS‐J: (1) frequency of exercise: “How often do you exercise in a week?” (days/week); and (2) mean duration of exercise: “About how long do you exercise in each session?” (min/session). Moreover, the total amount of exercise was estimated by multiplying the exercise frequency and mean exercise duration (min/week).^17^ In this study, participants with HTN who engaged in at least 30 min per session of exercise every day (7 days/week) or 180 min per week were considered to meet the current recommendation for exercise of the Japanese HTN management guidelines (2019).^6^

### 2.6 Statistical analyses

The proportion of patients who met the HTN management guidelines (i.e., engaging in exercise for at least 30 min daily or 180 min weekly) stratified by gender, age category (20–64, 65–74, and ≥75 years old), and BP level, were examined by pooled analyses of data from 2013 to 2018. These age categories were determined based on the classifications used in the HTN management guidelines.^6^ Moreover, we separately presented the proportions of participants exercising for at least 30 min daily or 180 min weekly as a sensitivity analysis. Differences in values between the stratified groups were assessed using the chi‐squared test or Fisher exact test for categorical data and Student's t‐test, one‐way analysis of variance (ANOVA), Mann–Whitney U test, or Kruskal–Wallis test for continuous variables, depending on the nature of the data. Additionally, the associations of the total amount of exercise duration (in units of 10 min per day increase) with systolic and diastolic BP (mmHg) were examined through multiple linear regression, stratified by whether the participants were taking antihypertensive drugs or not. Three multivariable models were used for this analysis: Model 1 was crude, Model 2 was adjusted for age and gender, and Model 3 was further adjusted for BMI.

Furthermore, adherence to the HTN management guidelines was compared among participants with different medical statuses (participants without HTN, those on antihypertensive medications, and those not on antihypertensive medications), with estimation of prevalence ratios (PR) by multivariable Poisson regression. This method was used because the prevalence of meeting the guidelines was over 10%.^20^ Model 1 was crude, Model 2 was adjusted for age, gender, and BMI, and Model 3 was further adjusted for the medication status for DLP and DM. All statistical analyses were performed using IBM SPSS Statistics version 28 (SPSS Inc., Chicago, IL, US), and the statistical significance level was set at *p* < 0.05.

## 3 RESULTS

**FIGURE 1:**
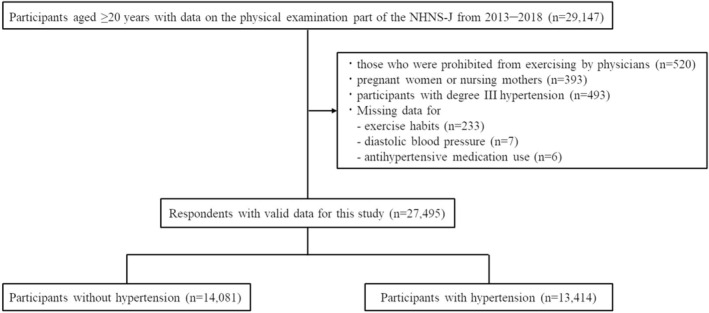
Participants flow. NHNS‐J, National Health and Nutrition Survey of Japan. Degree III hypertension was defined as systolic BP ≥180 and diastolic BP ≥110 mmHg according to the Japanese society of hypertension management guidelines.

Between 2013 and 2018, 29,147 individuals aged ≥20 years participated in the physical examination conducted as part of the NHNS‐J. A flowchart of the participant selection process is shown in Figure 1. After excluding 1652 participants, 27,495 participants were included in the analysis. Among them, 13,414 (48.8%) and 14,081 (51.2%) participants were classified into the hypertensive (i.e., >140/90 mmHg or taking antihypertensive drugs) and nonhypertensive (Figure 1) groups, respectively. Compared to participants without HTN, those with HTN were older, more likely to be men, more likely to have BMI ≥25 kg/m^2^, and had a greater exercise frequency and duration per week (Table 1). The proportion of those taking medication for DLP and DM was higher in participants with HTN than in those without HTN.

**TABLE 1:** Pooled characteristics of NHNS‐J participants from 2013 to 2018.

	Overall	Non‐HTN	HTN					
				Subcategories by BP level				
			HTN (total)	SBP<120 and DBP < 80	SBP 120–129 and DBP < 80	SBP 130–139 or DBP 80–89	SBP 140–159 or DBP 90–99	SBP 160–179 or DBP100–109
*n*	27,495	14,081	13,414	511	945	2523	7421	2014
Gender								
Men, *n* (%)	11,420 (41.5)	4966 (35.3)	6454 (48.1)	220 (43.1)	405 (42.9)	1157 (45.9)	3632 (48.9)	1040 (51.6)
Women, *n* (%)	16,075 (58.5)	9115 (64.7)	6960 (51.9)	291 (56.9)	540 (57.1)	1366 (54.1)	3789 (51.1)	974 (48.4)
Age (years), *n* (%)	60.5 (16.1)	53.2 (16.2)	68.2 (11.7)	71.2 (11)	72.7 (9.2)	71 (9.6)	66.6 (12.3)	67.5 (11.9)
20–64 years, *n* (%)	14,278 (51.9)	10,002 (71)	4276 (31.9)	117 (22.9)	154 (16.3)	573 (22.7)	2720 (36.7)	712 (35.4)
65–74 years, *n* (%)	7676 (27.9)	2715 (19.3)	4961 (37.0)	188 (36.8)	374 (39.6)	1002 (39.7)	2688 (36.2)	709 (35.2)
≥75 years, *n* (%)	5541 (20.2)	1364 (9.7)	4177 (31.1)	206 (40.3)	417 (44.1)	948 (37.6)	2013 (27.1)	593 (29.4)
BMI	23.1 (3.6)	22.2 (3.3)	24.1 (3.6)	23.5 (3.3)	23.7 (3.4)	24.3 (3.5)	24 (3.7)	24.2 (3.8)
<25 kg/m^2^, *n* (%)	20,248 (73.6)	11,592 (82.3)	8656 (64.5)	369 (72.2)	643 (68)	1572 (62.3)	4782 (64.4)	1290 (64.1)
≥25 kg/m^2^, *n* (%)	7208 (26.2)	2468 (17.5)	4740 (35.3)	140 (27.4)	302 (32)	947 (37.5)	2629 (35.4)	722 (35.8)
Missing, *n* (%)	39 (0.1)	21 (0.1)	18 (0.1)	2 (0.4)	0 (0)	4 (0.2)	10 (0.1)	2 (0.1)
SBP (mmHg)	130.3 (17.4)	118.9 (11.6)	142.4 (14.0)	112.2 (6.4)	124.9 (2.9)	132.3 (5.6)	144.8 (8.1)	161.9 (10.5)
DBP (mmHg)	78.5 (10.6)	74.1 (8.2)	83.2 (10.9)	68.1 (7.4)	70.7 (6.5)	78 (7.7)	85.1 (9)	92.3 (10.9)
Medication for HTN								
Yes, *n* (%)	8098 (29.5)	0 (0)	8098 (60.4)	511 (100)	945 (100)	2523 (100)	3270 (44.1)	849 (42.2)
No, *n* (%)	19,397 (70.5)	14,081 (100)	5316 (39.6)	0 (0)	0 (0)	0 (0)	4151 (55.9)	1165 (57.8)
Medication for dyslipidemia								
Yes, *n* (%)	4959 (18.0)	1298 (9.2)	3661 (27.3)	202 (39.5)	362 (38.3)	941 (37.3)	1727 (23.3)	429 (21.3)
No, *n* (%)	22,510 (81.9)	12,779 (90.8)	9731 (72.5)	308 (60.3)	579 (61.3)	1575 (62.4)	5687 (76.6)	1582 (78.6)
Missing, *n* (%)	26 (0.1)	4 (0.0)	22 (0.2)	1 (0.2)	4 (0.4)	7 (0.3)	7 (0.1)	3 (0.1)
Medication for diabetes mellitus								
Yes, *n* (%)	1993 (7.2)	467 (3.3)	1526 (11.4)	80 (15.7)	154 (16.3)	374 (14.8)	735 (9.9)	183 (9.1)
No, *n* (%)	22,821 (83.0)	12,184 (86.5)	10,637 (79.3)	383 (75.0)	717 (75.9)	1916 (75.9)	5994 (80.8)	1627 (80.8)
Missing, *n* (%)	2681 (9.8)	1430 (10.2)	1251 (9.3)	48 (9.4)	74 (7.8)	233 (9.2)	692 (9.3)	204 (10.1)
Total exercise time per week (min/week)^a^	30 (0–180)	0 (0–160)	60 (0–210)	70 (0–210)	60 (0–210)	60 (0–210)	60 (0–210)	40 (0–210)
Exercise frequency (days/week)	1 (0–4)	0 (0–3)	1 (0–5)	2 (0–5)	1 (0–5)	2 (0–5)	1 (0–5)	1 (0–5)
0 day/week, *n* (%)	13,195 (48.0)	7330 (52.1)	5865 (43.7)	201 (39.3)	393 (41.6)	1083 (42.9)	3249 (43.8)	939 (46.6)
1 day/week, *n* (%)	2450 (8.9)	1387 (9.9)	1063 (7.9)	36 (7.0)	86 (9.1)	163 (6.5)	622 (8.4)	156 (7.7)
2 days/week, *n* (%)	2110 (7.7)	1078 (7.7)	1032 (7.7)	50 (9.8)	70 (7.4)	196 (7.8)	583 (7.9)	133 (6.6)
3 days/week, *n* (%)	2102 (7.6)	1007 (7.2)	1095 (8.2)	41 (8.0)	92 (9.7)	225 (8.9)	604 (8.1)	133 (6.6)
4 days/week, *n* (%)	1174 (4.3)	551 (3.9)	623 (4.6)	30 (5.9)	32 (3.4)	122 (4.8)	345 (4.6)	94 (4.7)
5 days/week, *n* (%)	1445 (5.3)	708 (5)	737 (5.5)	31 (6.1)	50 (5.3)	144 (5.7)	407 (5.5)	105 (5.2)
6 days/week, *n* (%)	596 (2.2)	260 (1.8)	336 (2.5)	11 (2.2)	21 (2.2)	57 (2.3)	185 (2.5)	62 (3.1)
7 days/week, *n* (%)	4423 (16.1)	1760 (12.5)	2663 (19.9)	111 (21.7)	201 (21.3)	533 (21.1)	1426 (19.2)	392 (19.5)
Exercise duration (min/session)	60 (30–90)	60 (30–90)	60 (30–90)	60 (30–90)	60 (30–90)	60 (30–90)	60 (30–90)	60 (30–90)
1–14 min/session, *n* (%)	751 (2.7)	351 (2.5)	400 (3.0)	15 (2.9)	34 (3.6)	86 (3.4)	220 (3.0)	45 (2.2)
15–29 min/session, *n* (%)	1281 (4.7)	581 (4.1)	700 (5.2)	40 (7.8)	49 (5.2)	145 (5.7)	376 (5.1)	90 (4.5)
30–59 min/session, *n* (%)	4395 (16.0)	1960 (13.9)	2435 (18.2)	94 (18.4)	185 (19.6)	459 (18.2)	1335 (18.0)	362 (18.0)
≥60 min/session, *n* (%)	7846 (28.5)	3864 (27.4)	3982 (29.7)	159 (31.1)	283 (29.9)	737 (29.2)	2228 (30.0)	575 (28.6)

*Note*: Data are presented as mean (SD), median (25th–75th percentile), or number (%).

Abbreviations: BMI, body mass index; BP, blood pressure; DBP, diastolic blood pressure; HTN, hypertension; NHNS‐J, National Health and Nutrition Survey of Japan; SBP, systolic blood pressure.

^a^ Total amount of exercise time was calculated by multiplying the frequency (days/week) and duration of each exercise session (min/session) as continuous valuables.

The characteristics of the participants with HTN stratified by BP grade levels are shown in Table 1. The proportion of men with a BMI ≥25 kg/m^2^ tended to increase from lower toward higher BP grade level groups, whereas participants tended to be younger in higher BP grade level groups than those in lower BP grade level groups. There were significant differences across all comparisons (*p* < 0.05) (Table 1), except for exercise duration. Specifically, the median exercise frequency among all BP categories was <3 times/week, and there were no significant differences in total exercise duration (min/week) and each exercise duration (min/session) across BP grade levels among participants with HTN (*p* = 0.22 for total exercise duration and *p* = 0.12 for exercise duration per session, by Kruskal–Wallis test).

**FIGURE 2:**
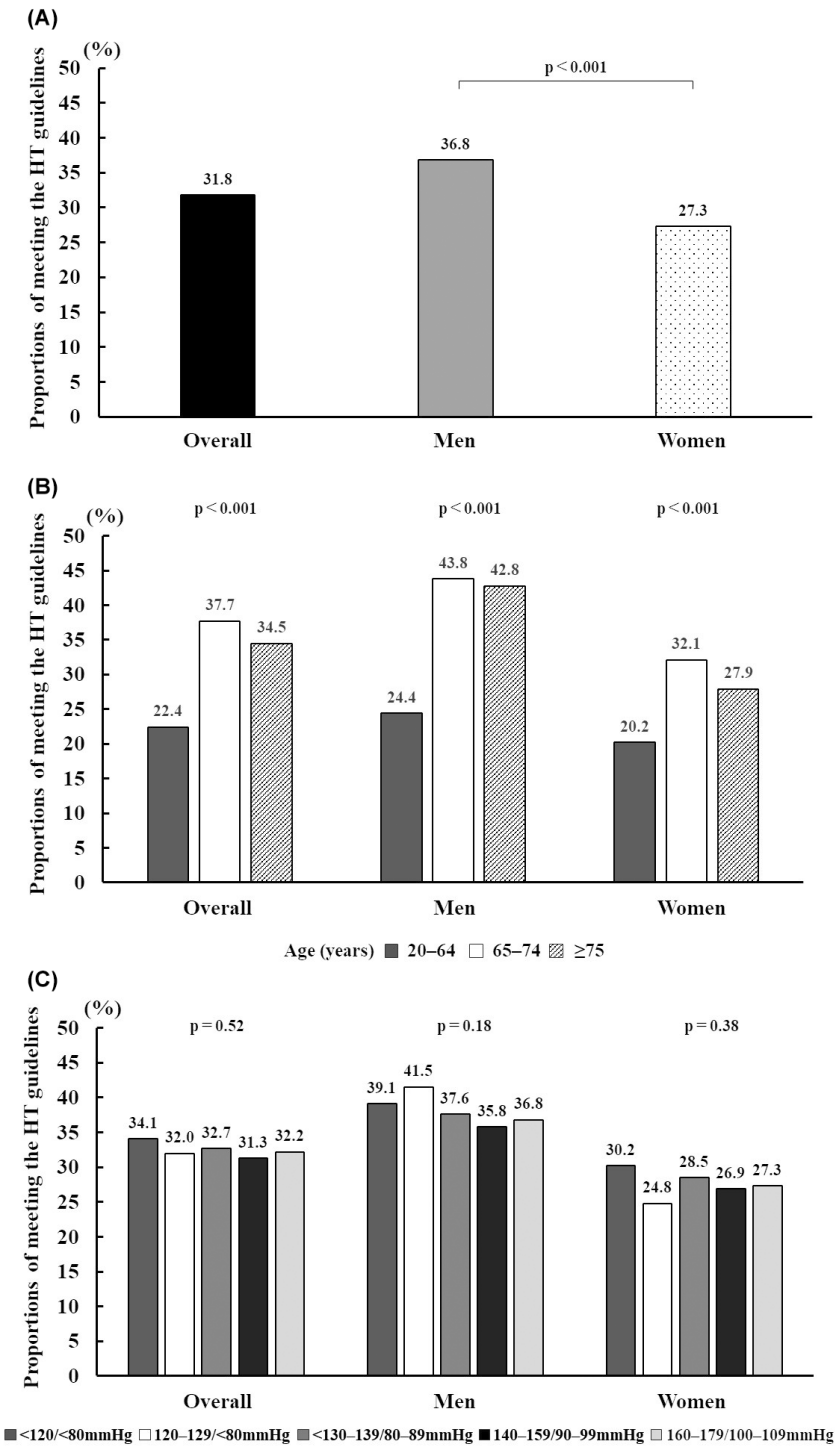
The proportion of those who met the HTN management guidelines stratified by (A) gender, (B) age and gender, and (C) blood pressure level.

Figure 2 shows the adherence to the regular exercise habits recommended by the HTN management guidelines among participants with HTN. In the pooled analyses of data obtained from 2013 to 2018, 31.8% (*n* = 4269) of the participants with HTN met the exercise recommendation, with a prevalence of 36.8% (*n* = 2372) and 27.3% (*n* = 1897) among men and women, respectively (Figure 2A).

Among participants with HTN, the 20–64 years age group showed the lowest number of participants met the HTN management guidelines for both genders (24.4% for men and 20.2% for women), followed by the ≥75 years of age group (42.8% in men and 27.9% in women) and the 65–74 years of age group (43.8% in men and 32.1% in women) (Figure 2B).

**FIGURE 3:**
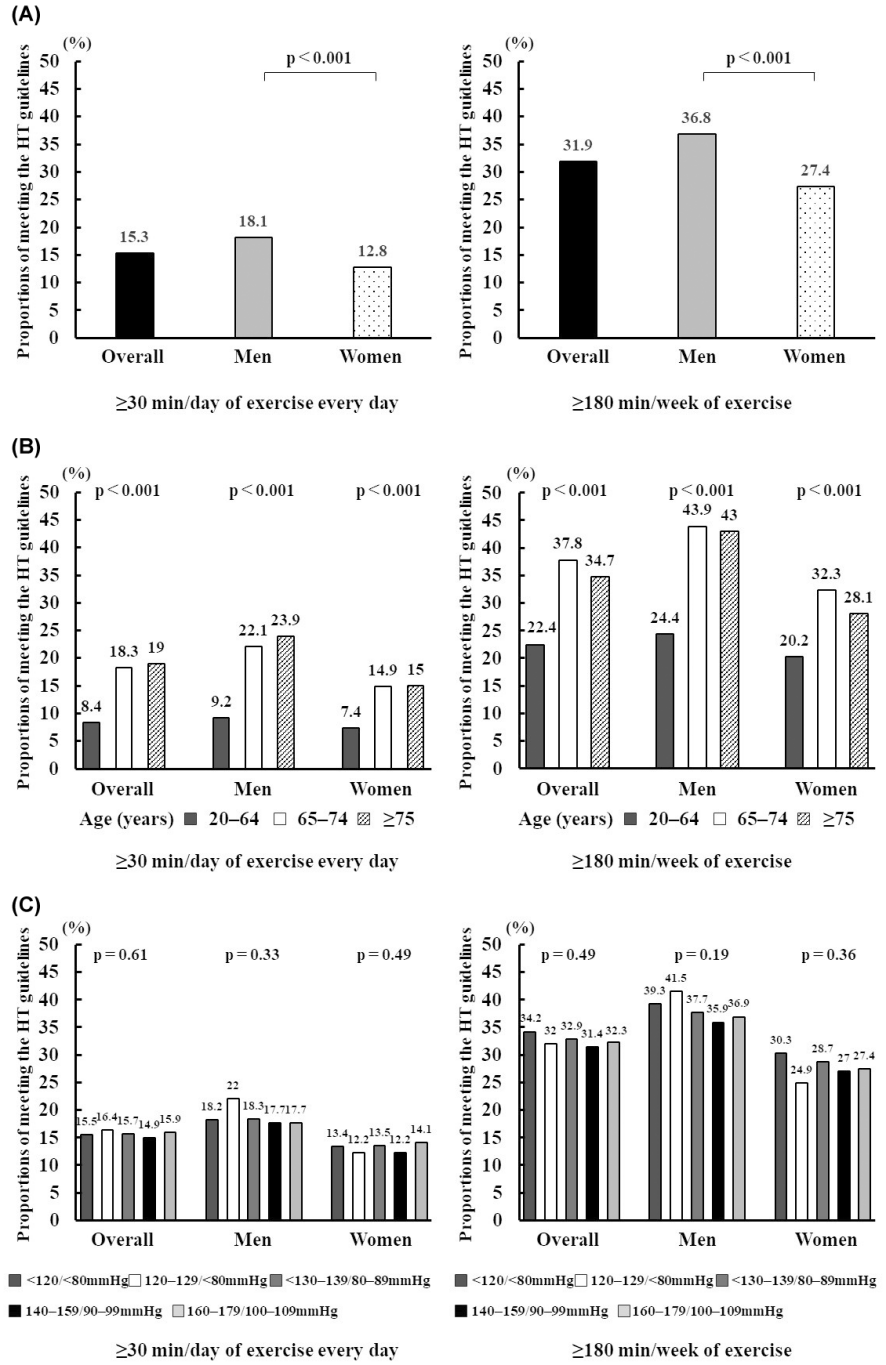
A sensitivity analysis of the proportions of participants exercising for at least 30 min daily (left side) or 180 min/week (right side). The proportion of those who met the HTN management guidelines for at least 30 min daily or 180 min/week, stratified by (A) gender, (B) age and gender, and (C) blood pressure level.

The proportion of participants with HTN meeting the HTN management guidelines stratified by BP level was 34.1% in the <120/80 group, 32.0% in the 120–129/<80 group, 32.7% in the 130–139/80–89 group, 31.3% in the 140–149/90–99 group, and 32.2% in the 150–159/100–109 group (Figure 2C). In all sensitivity analyses, the proportions of participants who exercised for 180 min/week were higher than those who exercised for at least 30 min/day as stratified by gender, age groups, and BP levels (Figure 3). Among participants aged 65–74 an ≥75 years, the proportion of those who met the exercise guideline of 180 min per week was twice higher than that of those who met the exercise guideline of at least 30 min every day. Among participants aged 20–64 years, the proportion of those who met the exercise guideline of 180 min per week was thrice higher than that of those who met the guideline of at least 30 min every day.

Regarding the relationship between total exercise duration and BP, longer exercise duration (10 min increase/day) was found to be significantly associated with lower systolic and diastolic BP among participants without drug therapy. Contrastingly, among participants with drug therapy, there were no significant associations between total exercise duration and BP levels (Table S1).

Furthermore, we compared the proportion of participants meeting the HTN management guidelines in the entire sample (*n* = 27,495) categorized into participants without HTN, participants with HTN using antihypertensive drug medications, and those who were not on antihypertensive drug medications (Table 2, Table S2, and Figure S1). The lowest mean age and proportion of men was observed among the participants without HTN (Table S2). In the crude analyses, adherence to the HTN management guidelines was significantly higher in participants with HTN than in those without (Table 2). However, after adjusting for age, gender, BMI, and use of other medications for DLP and DM, adherence to the exercise recommendation in HTN management guidelines did not significantly differ according to medical status regarding HTN (Table 2). Moreover, adherence to the exercise recommendation in HTN management guidelines significantly differed according to the use of medication for DM, but not DLP (Model 3).

**TABLE 2:** Comparisons of adherence to the HTN management guidelines regarding exercise according to medical status.

	Model 1			Model 2			Model 3		
	PR	95% CI	*p* value	PR	95% CI	*p* value	PR	95% CI	*p* value
Age, years	1.04	1.03–1.04	<0.001	1.04	1.03–1.04	<0.001	1.04	1.03–1.04	<0.001
Gender, female	0.66	0.62–0.71	<0.001	0.69	0.64–0.74	<0.001	0.70	0.65–0.75	<0.001
BMI, kg/m^2^	1.00	0.99–1.01	0.63	0.99	0.98–1.00	0.04	0.99	0.98–1.00	0.01
Status of HTN									
Non‐HTN	Reference	–	–	Reference	–	–	Reference	–	–
Participants with HTN without medical treatments	1.46	1.33–1.61	<0.001	1.07	0.97–1.18	0.18	1.07	0.97–1.19	0.15
Participants with HTN with medical treatments	1.79	1.66–1.94	<0.001	1.06	0.97–1.16	0.24	1.03	0.94–1.13	0.50
Taking antidyslipidemia medication									
Yes	1.37	1.26–1.48	<0.001	–	–	–	1.05	0.97–1.15	0.24
No	Reference	–	–	–	–	–	Reference	–	–
Taking antidiabetes medication									
Yes	1.66	1.49–1.85	<0.001	–	–	–	1.21	1.09–1.35	<0.001
No	Reference	–	–	–	–	–	Reference	–	–

*Note*: Model 1: crude model (no adjustment). Model 2: age, gender, and BMI were added to Model 1. Model 3: Status of taking medication for dyslipidemia and diabetes mellitus was added to Model 2.

Abbreviations: BMI, body mass index; CI, confidence interval; HTN, hypertension; PR, prevalence ratio.

## 4 DISCUSSION

We demonstrated that (1) only 31.8% of participants with HTN met the exercise recommendations in the HTN management guidelines; (2) the proportion of those who met the exercise recommendations was higher in men then in women; however, the proportion in men was not sufficiently high; (3) older adults with HTN aged ≥65 and ≥75 years engaged in exercise more than younger and middle‐aged adults with HTN (20–64 years old); and (4) the prevalence of exercise habits did not differ significantly across BP levels. Our findings suggest that further promotion of exercise is required in hypertensive populations in both genders and across all BP levels. In clinical practice, younger and middle‐aged adults with HTN should be targeted when promoting healthy lifestyle modifications.

From the psychological perspective of promoting physical activity, factors such as awareness, knowledge, beliefs, and behavioral intentions toward guideline recommendations influence behavioral changes concerning exercising.^21^ Doctors' knowledge is reflected in their clinical practice and could facilitate behavioral changes in patients through health education in clinical settings,^22^, ^23^ and, in Japan, ≈90% of physicians answered that they were aware of the Japanese HTN management guidelines.^24^ Indeed, the prevalence rate of meeting the HTN management guidelines in participants with HTN seemed to be higher than that in participants without HTN in the crude model. However, since the proportion of older participants and men, who were more engaged in exercise than younger participants and women, was higher among participants using antihypertensives than in the other groups, the association between medical status and adherence to the guidelines disappeared after adjustment for age, gender, and BMI. Despite the Japanese HTN management guidelines recommending exercise to all patients with HTN,^6^ the low rate of meeting the exercise recommendation among participants with HTN suggested insufficient health education on exercise in clinical settings for patients with HTN.^25^ Further dissemination of exercise therapy after future revisions of the Japanese HTN management guidelines is required by physicians to achieve better BP control and prevent cardiovascular diseases among patients with HTN.^1^, ^2^, ^3^, ^4^, ^5^

Furthermore, participants taking medication for DM were more engaged in exercise in the fully adjusted model, whereas those who were taking medication for DLP did not engage in exercise compared with those who were not taking medication. These findings suggested that the intention of lifestyle modification may differ among patients with noncommunicable diseases (NCDs), such as DM, DLP, and HTN.^26^, ^27^ Since health education would be useful to encourage lifestyle modification, further understanding of the intention and readiness for exercise among patients with different NCDs is required to facilitate better health education in clinical settings.^6^, ^25^

We observed that 31.8% of Japanese participants with HTN exercised regularly to meet the HTN management guidelines. The prevalence of regular exercise (e.g., walking and biking) among patients with HTN was 44.9% in Ethiopia^11^ and 51.9% in China.^13^ However, these studies considered an adequate exercise habit as engaging in 30 min of physical activity in 8–14 days,^11^ corresponding to approximately 120 min/week, or any exercise in 4 days per week.^13^ Contrastingly, the present study assessed the prevalence of engaging in exercise for at least 30 min every day or 180 min/week, based on the Japanese HTN management guidelines.^6^ Therefore, the prevalence of exercise habits in patients with HTN in Ethiopia^11^ and China^13^ may be overestimated compared with our results. Furthermore, Motlagh et al. reported only a 24.5% prevalence of exercise habits in Iranian patients with HTN.^12^ However, this study had some limitations, that is, >80% of the participants were aged >50 years, 62% were illiterate, and 90% had a poor income status.^12^ Aging and low socioeconomic status might have led to poor health literacy about HTN management,^28^ leading to underestimation of the prevalence of exercise habits.^25^ Further studies are required to examine the prevalence of exercise habits for HTN management in low‐ and middle‐income countries as well as high‐income countries.

Regarding age groups, the 20–64 years age group showed the lowest proportion of participants with adequate exercise habits. Since exercise is often considered a leisure‐time activity in high‐income countries,^29^ lack of time is a major barrier to engaging in exercise,^30^ especially in young adults and women.^31^ Indeed, more than one‐third of Japanese adults aged ≥20 years reported feeling like their jobs or household chores hamper engagement in exercise.^32^ Accordingly, the proportion of those performing exercise for 180 min per week (e.g., “weekend warriors” who engaged in exercise only over the weekend were also included here) was higher than those who exercised for at least 30 min daily. Our findings indicate that accommodating 180 min of exercise per week would be more achievable than daily exercise among patients with HTN, especially in younger and middle‐aged adults. Although the health benefits of “weekend warriors” have been reported,^33^ it remains unclear whether patients with HTN who engage in exercise regimen experience similar benefits. Currently, the Japanese HTN management guidelines recommend engaging in exercise for at least 30 min daily or 180 min weekly, with the recommendation for 180 min weekly exercise being newly added in the HTN management guidelines 2019^6^; however, the rate of achievement for each recommendation may differ. Therefore, the effect of each recommendation (i.e., at least 30 min daily or 180 min weekly) on health outcomes should be further examined as long as these combined recommendations continue.

This study has several limitations. First, the response rates for physical examination in the NHNS‐J ranged from 49.3%–56.9%,^18^ which might have resulted in selection bias. Given that more health‐conscious individuals and older people who perform exercise may have participated in the survey, our results may have been overestimated. Second, although physical activity includes exercise and daily activity (e.g., occupational, physical activity, transportation, and household chores),^34^, ^35^ we only mentioned the exercise aspect of physical activity. In this regard, Japanese HTN management guidelines mainly refer to exercise rather than physical activity. However, even those who do not exercise regularly may engage in sufficient daily physical activity, which could provide health benefits.^36^, ^37^ Further studies are warranted to investigate the total amount of daily physical activity among patients with HTN who have exercise habits. Third, in the NHNS‐J, since BP was measured by medical staff at designated community centers, the white coat phenomenon might affect the BP measurements. The white coat phenomenon might cause BP measurements to be overestimated compared to home BP monitoring, especially in participants without antihypertensive treatment (untreated).^6^, ^38^ Finally, although we assessed the frequency and duration of exercise, we did not have detailed information on the intensity^39^ and types of exercise, with the intensity of exercise being newly noted in the current HTN management guidelines.^6^

## 5 CONCLUSION

In a nationally representative sample from Japan, the proportion of participants with HTN meeting the exercise recommendations in the HTN management guidelines was only 31.8%. Moreover, although all patients with HTN were recommended to engage in exercise by the HTN management guidelines, the prevalence of engaging in adequate exercise did not significantly differ between participants with and without HTN, regardless of age, gender, BMI, and medication status for DLP and DM. Exercise therapy recommended in the HTN management guidelines should be further promoted in clinical settings.

## FUNDING INFORMATION

This research was supported by AMED under Grant Number JP22rea522002 and by MHLW Program (Grant Number JPMH22FA1004).

## CONFLICT OF INTEREST STATEMENT

Authors declare no Conflict of Interests for this article.

## ETHICS APPROVAL STATEMENT

This was a secondary analysis of the anonymized data obtained by the MHLW. The use of individual raw data from the NHNS‐J from 2013 to 2018 was approved by the MHLW through official application procedures under Article 33 of the Japanese Statistics Act. Furthermore, the institutional review board waived the requirement of ethical review for this study.

## PATIENT CONSENT STATEMENT

Not applicable because of a secondary analysis of the anonymized data obtained by the Ministry of Health, Labor, and Welfare.

## CLINICAL TRIAL REGISTRATION

Not applicable.

## Supporting information


Appendix S1.

## Data Availability

The data that support the findings of this study are available from the Ministry of Health, Labor, and Welfare (MHLW). Restrictions apply to the availability of these data, which were under Article 33 of the Japanese Statistics Act.
