# Supplementary material for: Descriptive epidemiology of prevalence of exercise habits among participants with hypertension: The National Health and Nutrition Survey 2013–2018
Source: J Gen Fam Med. 2024 Mar 1;25(3):128–39. doi: 10.1002/jgf2.683 (PMC11065153; doi:10.1002/jgf2.683)
Supplement: Supplementary file 1 — Appendix S1. [file JGF2-25-128-s001.pdf]

The supplementary information

- Supplementary Figure S1 The proportion of healthy participants with normal blood pressure meeting the hypertension (HTN) management guidelines stratified by A) sex, and B) age and sex category.
- Supplementary Table S1. Associations of total exercise duration with systolic and diastolic blood pressure, stratified by whether the participants were taking antihypertensive drugs or not.
- Supplementary Table S2. Participant characteristics stratified by medical status.

Supplementary Figure S1 The proportion of healthy participants with normal blood pressure meeting the hypertension (HTN) management guidelines stratified by A) sex, and B) age and sex category.

A

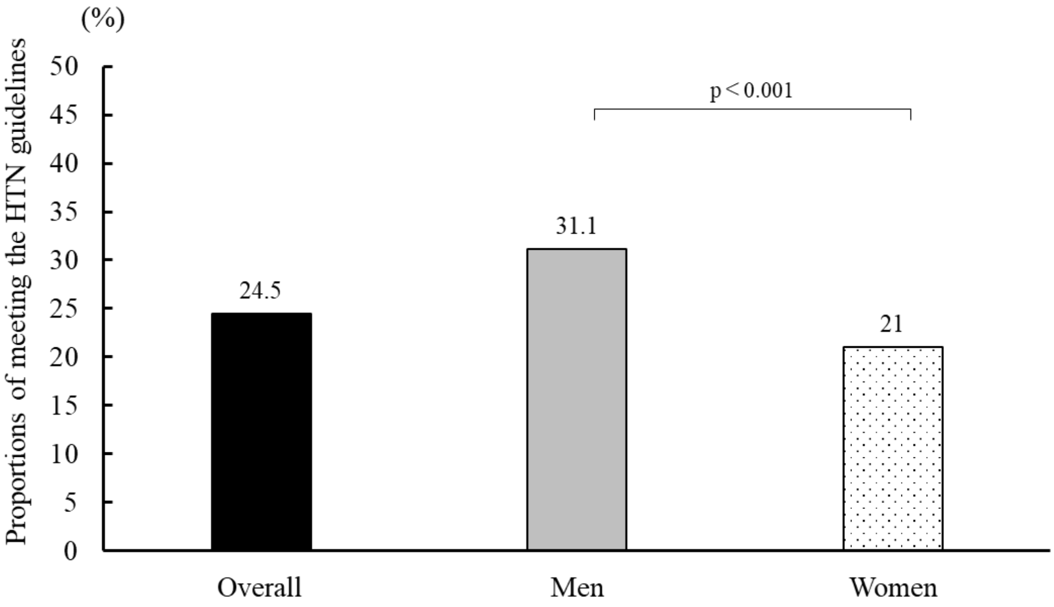

B

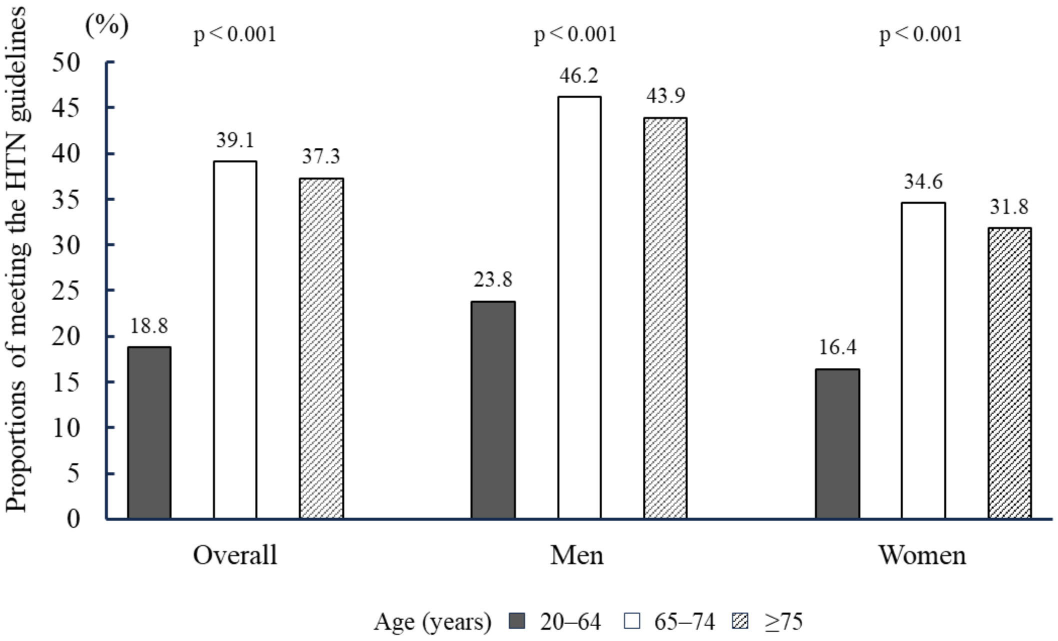

Supplementary Table S1. Associations of total exercise duration with systolic and diastolic blood pressure, stratified by whether the participants were taking antihypertensive drugs or not.

	Systolic blood pressure								
	Model 1			Model 2			Model 3		
	β	95%CI	p value	β	95%CI	p value	β	95%CI	p value
Participants with drug treatment									
Exercise duration (10min increase per day)	0.08	(-0.01, -0.17)	0.09	0.04	(-0.05, 0.13)	0.37	0.05	(-0.04, 0.15)	0.25
Participants without drug treatment									
Exercise duration (10min increase per day)	0.35	(0.28, 0.42)	<.001	-0.09	(-0.15, -0.03)	0.005	-0.08	(-0.14, -0.02)	0.01
	Diastolic blood pressure								
	Model 1			Model 2			Model 3		
	β	95%CI	p value	β	95%CI	p value	β	95%CI	p value
Participants with drug treatment									
Exercise duration (10min increase per day)	-0.05	(-0.11, 0.02)	0.183	-0.01	(-0.07, 0.06)	0.88	0.01	(-0.05, 0.07)	0.75
Participants without drug treatment									
Exercise duration (10min increase per day)	0.07	(0.03, 0.11)	0.001	-0.09	(-0.13, -0.05)	<.001	-0.08	(-0.12, -0.04)	<.001

β, Unstandardized coefficients; CI: Confidence interval.
Model 1: crude model (no adjustment)
Model 2: age and sex were added into Model 1.
Model 3: body mass index, systolic blood pressure and diastolic blood pressure were added into Model 2.

Supplementary Table S2. Participant characteristics stratified by medical status.

	Non-HTN (n=14 081)	Participants with HTN without medical treatments (n=5316)	Participants with HTN with medical treatments (n=8098)	P value
Age (years)	53.2 (16.2)	63.4 (12.7)	71.3 (9.8)	<0.001
Men, n (%)	4966 (35.3)	2652 (49.9)	3802 (46.9)	<0.001
Women, n (%)	9115 (64.7)	2664 (50.1)	4296 (53.1)	
Systolic BP (mmHg)	118.9 (11.6)	147.6 (11.5)	139 .0 (14.5)	<0.001
Diastolic BP (mmHg)	74.1 (8.2)	88.5 (9.2)	79.7 (10.4)	<0.001
BMI (kg/m^2)	22.2 (3.3)	23.7 (3.7)	24.3 (3.6)	<0.001
Taking anti-dyslipidemia medication, n (%)	1298 (9.2)	666 (12.5)	2995 (37.0)	<0.001
Taking anti-diabetes medication, n (%)	467 (3.3)	282 (5.3)	1244 (15.4)	<0.001
Proportion of meeting the JSH2019 guidelines, n (%)	3453 (24.5)	1564 (29.4)	2705 (33.4)	<0.001

Data are presented as the mean (SD) or number (%)
HTN; hypertension, BP; blood pressure, BMI; body mass index, JSH; Japanese society of hypertension.
